# Supplementary material for: Modeling glioblastoma heterogeneity as a dynamic network of cell states
Source: Mol Syst Biol. 2021 Sep 16;17(9):e10105. doi: 10.15252/msb.202010105 (PMC8444284; doi:10.15252/msb.202010105)
Supplement: Supplementary file 6 — Source Data for Figure 5 [file MSB-17-e10105-s004.zip › Figure5A_sourcedata/GSEA_3017/nosigdb_stateF.GseaPreranked.1622795015644/RICHARDS_ZHONG_NATURE_2018_ASTROCYTES_UPREG.html]

Details for gene set RICHARDS\_ZHONG\_NATURE\_2018\_ASTROCYTES\_UPREG[GSEA]

|  || Dataset | state33017 |
| Phenotype | NoPhenotypeAvailable |
| Upregulated in class | na\_pos |
| GeneSet | RICHARDS\_ZHONG\_NATURE\_2018\_ASTROCYTES\_UPREG |
| Enrichment Score (ES) | 0.52110314 |
| Normalized Enrichment Score (NES) | 3.6072187 |
| Nominal p-value | 0.0 |
| FDR q-value | 0.0 |
| FWER p-Value | 0.0 |
Table: GSEA Results Summary

  

Fig 1: Enrichment plot: RICHARDS\_ZHONG\_NATURE\_2018\_ASTROCYTES\_UPREG      
 Profile of the Running ES Score & Positions of GeneSet Members on the Rank Ordered List

  

| PROBE | GENE SYMBOL | GENE\_TITLE | RANK IN GENE LIST | RANK METRIC SCORE | RUNNING ES | CORE ENRICHMENT || 1 | MT3 |  |  | 1 | 2.229 | 0.0241 | Yes |
| 2 | CLU |  |  | 5 | 1.852 | 0.0431 | Yes |
| 3 | SPARCL1 |  |  | 6 | 1.804 | 0.0630 | Yes |
| 4 | ATP1B2 |  |  | 9 | 1.759 | 0.0815 | Yes |
| 5 | HOPX |  |  | 14 | 1.345 | 0.0944 | Yes |
| 6 | CST3 |  |  | 15 | 1.273 | 0.1085 | Yes |
| 7 | LGALS3 |  |  | 16 | 1.261 | 0.1224 | Yes |
| 8 | APOE |  |  | 17 | 1.244 | 0.1361 | Yes |
| 9 | S100B |  |  | 20 | 1.167 | 0.1480 | Yes |
| 10 | RAMP1 |  |  | 24 | 1.069 | 0.1584 | Yes |
| 11 | SLC1A3 |  |  | 26 | 1.058 | 0.1696 | Yes |
| 12 | CSPG5 |  |  | 30 | 1.044 | 0.1797 | Yes |
| 13 | LRRC17 |  |  | 31 | 1.044 | 0.1912 | Yes |
| 14 | ITPR2 |  |  | 34 | 1.024 | 0.2016 | Yes |
| 15 | PLTP |  |  | 35 | 1.020 | 0.2128 | Yes |
| 16 | DKK3 |  |  | 37 | 0.994 | 0.2233 | Yes |
| 17 | IFITM3 |  |  | 38 | 0.987 | 0.2342 | Yes |
| 18 | SLC4A4 |  |  | 40 | 0.980 | 0.2445 | Yes |
| 19 | F3 |  |  | 41 | 0.978 | 0.2553 | Yes |
| 20 | EDNRB |  |  | 44 | 0.957 | 0.2649 | Yes |
| 21 | GFAP |  |  | 47 | 0.953 | 0.2745 | Yes |
| 22 | CXCL14 |  |  | 51 | 0.931 | 0.2833 | Yes |
| 23 | MLC1 |  |  | 58 | 0.896 | 0.2904 | Yes |
| 24 | SLC1A2 |  |  | 63 | 0.857 | 0.2979 | Yes |
| 25 | TSC22D4 |  |  | 64 | 0.855 | 0.3073 | Yes |
| 26 | GLUL |  |  | 69 | 0.837 | 0.3147 | Yes |
| 27 | AGT |  |  | 72 | 0.820 | 0.3228 | Yes |
| 28 | SERPINB6 |  |  | 83 | 0.791 | 0.3267 | Yes |
| 29 | AQP4 |  |  | 84 | 0.789 | 0.3354 | Yes |
| 30 | CBR1 |  |  | 87 | 0.779 | 0.3430 | Yes |
| 31 | UBL3 |  |  | 88 | 0.778 | 0.3516 | Yes |
| 32 | PON2 |  |  | 93 | 0.764 | 0.3581 | Yes |
| 33 | HES5 |  |  | 102 | 0.749 | 0.3626 | Yes |
| 34 | LUZP2 |  |  | 106 | 0.742 | 0.3693 | Yes |
| 35 | B2M |  |  | 110 | 0.736 | 0.3760 | Yes |
| 36 | GNG7 |  |  | 115 | 0.726 | 0.3821 | Yes |
| 37 | NPNT |  |  | 117 | 0.721 | 0.3896 | Yes |
| 38 | NCAN |  |  | 138 | 0.686 | 0.3876 | Yes |
| 39 | TP53I3 |  |  | 139 | 0.686 | 0.3952 | Yes |
| 40 | TSPAN7 |  |  | 148 | 0.671 | 0.3987 | Yes |
| 41 | DDR1 |  |  | 159 | 0.649 | 0.4011 | Yes |
| 42 | ITM2C |  |  | 160 | 0.649 | 0.4083 | Yes |
| 43 | DTNA |  |  | 176 | 0.617 | 0.4079 | Yes |
| 44 | ADGRG1 |  |  | 184 | 0.606 | 0.4113 | Yes |
| 45 | C1orf61 |  |  | 202 | 0.587 | 0.4096 | Yes |
| 46 | ENHO |  |  | 203 | 0.586 | 0.4161 | Yes |
| 47 | BTBD17 |  |  | 217 | 0.578 | 0.4162 | Yes |
| 48 | GPR37L1 |  |  | 220 | 0.576 | 0.4216 | Yes |
| 49 | PDLIM5 |  |  | 223 | 0.572 | 0.4270 | Yes |
| 50 | TM7SF2 |  |  | 225 | 0.570 | 0.4328 | Yes |
| 51 | HTRA1 |  |  | 229 | 0.568 | 0.4376 | Yes |
| 52 | LIFR |  |  | 239 | 0.562 | 0.4395 | Yes |
| 53 | PHYHIPL |  |  | 245 | 0.557 | 0.4433 | Yes |
| 54 | PBXIP1 |  |  | 250 | 0.554 | 0.4475 | Yes |
| 55 | S100A13 |  |  | 261 | 0.544 | 0.4487 | Yes |
| 56 | GPR137B |  |  | 265 | 0.541 | 0.4532 | Yes |
| 57 | HMGCS1 |  |  | 271 | 0.537 | 0.4567 | Yes |
| 58 | SLC25A18 |  |  | 280 | 0.525 | 0.4587 | Yes |
| 59 | BCAN |  |  | 302 | 0.510 | 0.4543 | Yes |
| 60 | PTPRZ1 |  |  | 310 | 0.505 | 0.4565 | Yes |
| 61 | HES1 |  |  | 320 | 0.501 | 0.4577 | Yes |
| 62 | PSAT1 |  |  | 329 | 0.496 | 0.4594 | Yes |
| 63 | OLFM2 |  |  | 343 | 0.490 | 0.4586 | Yes |
| 64 | ITGAV |  |  | 344 | 0.490 | 0.4640 | Yes |
| 65 | ACSBG1 |  |  | 345 | 0.490 | 0.4694 | Yes |
| 66 | ABAT |  |  | 362 | 0.483 | 0.4670 | Yes |
| 67 | LIPA |  |  | 364 | 0.483 | 0.4719 | Yes |
| 68 | ID2 |  |  | 366 | 0.483 | 0.4767 | Yes |
| 69 | GATM |  |  | 375 | 0.480 | 0.4782 | Yes |
| 70 | CTSL |  |  | 376 | 0.479 | 0.4835 | Yes |
| 71 | SEMA6A |  |  | 381 | 0.478 | 0.4868 | Yes |
| 72 | LRRTM3 |  |  | 387 | 0.476 | 0.4897 | Yes |
| 73 | COMT |  |  | 396 | 0.471 | 0.4911 | Yes |
| 74 | METTL7A |  |  | 403 | 0.469 | 0.4934 | Yes |
| 75 | SLC3A2 |  |  | 411 | 0.466 | 0.4952 | Yes |
| 76 | PRCP |  |  | 426 | 0.460 | 0.4935 | Yes |
| 77 | HSD17B12 |  |  | 453 | 0.449 | 0.4860 | Yes |
| 78 | TTYH1 |  |  | 457 | 0.448 | 0.4895 | Yes |
| 79 | EFHD2 |  |  | 464 | 0.445 | 0.4916 | Yes |
| 80 | KLHDC8A |  |  | 493 | 0.438 | 0.4830 | Yes |
| 81 | GJA1 |  |  | 495 | 0.436 | 0.4874 | Yes |
| 82 | HEY1 |  |  | 496 | 0.436 | 0.4922 | Yes |
| 83 | TIMP3 |  |  | 516 | 0.428 | 0.4878 | Yes |
| 84 | PEA15 |  |  | 521 | 0.427 | 0.4906 | Yes |
| 85 | CA12 |  |  | 522 | 0.426 | 0.4953 | Yes |
| 86 | LAMB2 |  |  | 524 | 0.425 | 0.4995 | Yes |
| 87 | ELOVL2 |  |  | 527 | 0.424 | 0.5032 | Yes |
| 88 | LGALS3BP |  |  | 528 | 0.424 | 0.5079 | Yes |
| 89 | TFPI |  |  | 531 | 0.421 | 0.5116 | Yes |
| 90 | GPM6B |  |  | 553 | 0.413 | 0.5061 | Yes |
| 91 | GRB14 |  |  | 566 | 0.409 | 0.5049 | Yes |
| 92 | CNN3 |  |  | 576 | 0.407 | 0.5050 | Yes |
| 93 | RAB31 |  |  | 580 | 0.404 | 0.5081 | Yes |
| 94 | SLC6A1 |  |  | 582 | 0.404 | 0.5120 | Yes |
| 95 | PLEKHB1 |  |  | 597 | 0.399 | 0.5098 | Yes |
| 96 | TNC |  |  | 613 | 0.393 | 0.5069 | Yes |
| 97 | SASH1 |  |  | 614 | 0.393 | 0.5112 | Yes |
| 98 | ARNTL |  |  | 617 | 0.393 | 0.5146 | Yes |
| 99 | SEMA6D |  |  | 637 | 0.388 | 0.5098 | Yes |
| 100 | ANOS1 |  |  | 641 | 0.387 | 0.5126 | Yes |
| 101 | GPRC5B |  |  | 653 | 0.385 | 0.5116 | Yes |
| 102 | SEZ6L |  |  | 657 | 0.383 | 0.5144 | Yes |
| 103 | ID4 |  |  | 659 | 0.383 | 0.5182 | Yes |
| 104 | ADD3 |  |  | 674 | 0.378 | 0.5156 | Yes |
| 105 | BCHE |  |  | 677 | 0.376 | 0.5188 | Yes |
| 106 | CCDC80 |  |  | 683 | 0.375 | 0.5206 | Yes |
| 107 | ZFYVE21 |  |  | 694 | 0.373 | 0.5199 | Yes |
| 108 | FABP7 |  |  | 701 | 0.371 | 0.5211 | Yes |
| 109 | FAM107A |  |  | 718 | 0.367 | 0.5175 | No |
| 110 | GNPTG |  |  | 734 | 0.363 | 0.5143 | No |
| 111 | MASP1 |  |  | 755 | 0.357 | 0.5087 | No |
| 112 | RIT2 |  |  | 756 | 0.357 | 0.5126 | No |
| 113 | LRP4 |  |  | 773 | 0.353 | 0.5089 | No |
| 114 | SLC15A2 |  |  | 784 | 0.351 | 0.5079 | No |
| 115 | C3orf70 |  |  | 794 | 0.348 | 0.5075 | No |
| 116 | FAM84B |  |  | 804 | 0.346 | 0.5070 | No |
| 117 | BDH2 |  |  | 806 | 0.346 | 0.5103 | No |
| 118 | DAG1 |  |  | 834 | 0.340 | 0.5012 | No |
| 119 | TSPAN6 |  |  | 838 | 0.340 | 0.5035 | No |
| 120 | RCN1 |  |  | 849 | 0.337 | 0.5024 | No |
| 121 | ZFP36L2 |  |  | 850 | 0.336 | 0.5061 | No |
| 122 | MSMO1 |  |  | 860 | 0.333 | 0.5055 | No |
| 123 | SDC3 |  |  | 863 | 0.333 | 0.5082 | No |
| 124 | ARHGEF26 |  |  | 885 | 0.328 | 0.5018 | No |
| 125 | CRYL1 |  |  | 891 | 0.327 | 0.5030 | No |
| 126 | ADCYAP1R1 |  |  | 913 | 0.324 | 0.4965 | No |
| 127 | PLEC |  |  | 914 | 0.324 | 0.5001 | No |
| 128 | GDPD2 |  |  | 921 | 0.323 | 0.5008 | No |
| 129 | NKAIN4 |  |  | 923 | 0.322 | 0.5038 | No |
| 130 | HES4 |  |  | 950 | 0.317 | 0.4949 | No |
| 131 | SLITRK2 |  |  | 967 | 0.315 | 0.4907 | No |
| 132 | FAT1 |  |  | 968 | 0.315 | 0.4942 | No |
| 133 | ALDH6A1 |  |  | 987 | 0.311 | 0.4890 | No |
| 134 | EMC2 |  |  | 1001 | 0.310 | 0.4862 | No |
| 135 | RGMA |  |  | 1010 | 0.308 | 0.4858 | No |
| 136 | SERPINE2 |  |  | 1030 | 0.304 | 0.4800 | No |
| 137 | SCRG1 |  |  | 1032 | 0.304 | 0.4829 | No |
| 138 | CPNE5 |  |  | 1038 | 0.302 | 0.4838 | No |
| 139 | CD9 |  |  | 1070 | 0.297 | 0.4723 | No |
| 140 | TSPAN3 |  |  | 1071 | 0.297 | 0.4756 | No |
| 141 | C1orf122 |  |  | 1081 | 0.296 | 0.4745 | No |
| 142 | DNER |  |  | 1104 | 0.292 | 0.4672 | No |
| 143 | SYPL1 |  |  | 1137 | 0.287 | 0.4551 | No |
| 144 | SNX3 |  |  | 1150 | 0.285 | 0.4525 | No |
| 145 | DOK5 |  |  | 1193 | 0.279 | 0.4354 | No |
| 146 | RGS20 |  |  | 1197 | 0.279 | 0.4371 | No |
| 147 | VCAM1 |  |  | 1266 | 0.272 | 0.4075 | No |
| 148 | NLGN3 |  |  | 1292 | 0.269 | 0.3985 | No |
| 149 | RFX4 |  |  | 1320 | 0.266 | 0.3885 | No |
| 150 | GLUD1 |  |  | 1334 | 0.264 | 0.3852 | No |
| 151 | TRIM47 |  |  | 1353 | 0.262 | 0.3795 | No |
| 152 | SLCO1C1 |  |  | 1372 | 0.260 | 0.3737 | No |
| 153 | ABCD3 |  |  | 1381 | 0.259 | 0.3728 | No |
| 154 | ABHD3 |  |  | 1382 | 0.259 | 0.3756 | No |
| 155 | EEPD1 |  |  | 1397 | 0.257 | 0.3718 | No |
| 156 | WSCD1 |  |  | 1401 | 0.256 | 0.3731 | No |
| 157 | MEIS1 |  |  | 1407 | 0.256 | 0.3736 | No |
| 158 | TEX264 |  |  | 1419 | 0.255 | 0.3711 | No |
| 159 | NOTCH2 |  |  | 1420 | 0.255 | 0.3739 | No |
| 160 | EFEMP2 |  |  | 1428 | 0.255 | 0.3734 | No |
| 161 | BBS2 |  |  | 1472 | 0.250 | 0.3556 | No |
| 162 | ZFP36L1 |  |  | 1590 | -0.284 | 0.3027 | No |
| 163 | DCXR |  |  | 1689 | -0.315 | 0.2593 | No |
| 164 | LGALS1 |  |  | 1799 | -0.362 | 0.2111 | No |
| 165 | FABP5 |  |  | 2033 | -0.523 | 0.1053 | No |
Table: GSEA details [plain text format]

  

Fig 2: RICHARDS\_ZHONG\_NATURE\_2018\_ASTROCYTES\_UPREG: Random ES distribution      
 Gene set null distribution of ES for **RICHARDS\_ZHONG\_NATURE\_2018\_ASTROCYTES\_UPREG**
